# Full-Length Dystrophin Restoration via Targeted Exon Addition in DMD-Patient Specific iPSCs and Cardiomyocytes

**DOI:** 10.3390/ijms23169176

**Published:** 2022-08-16

**Authors:** Rou Xiao, Miaojin Zhou, Peiyun Wang, Baitao Zeng, Lingqian Wu, Zhiqing Hu, Desheng Liang

**Affiliations:** Center for Medical Genetics, School of Life Sciences, Central South University, Changsha 410078, China

**Keywords:** Duchenne muscular dystrophy, induced pluripotent stem cells, targeted exon addition, cardiomyocytes, full-length dystrophin

## Abstract

Duchenne muscular dystrophy (DMD) is the most common fatal muscle disease, with an estimated incidence of 1/3500–1/5000 male births, and it is associated with mutations in the X-linked *DMD* gene encoding dystrophin, the largest known human gene. There is currently no cure for DMD. The large size of the *DMD* gene hampers exogenous gene addition and delivery. The genetic correction of DMD patient-derived induced pluripotent stem cells (DMD-iPSCs) and differentiation into suitable cells for transplantation is a promising autologous therapeutic strategy for DMD. In this study, using CRISPR/Cas9, the full-length dystrophin coding sequence was reconstructed in an exon-50-deleted DMD-iPSCs by the targeted addition of exon 50 at the junction of exon 49 and intron 49 via homologous-directed recombination (HDR), with a high targeting efficiency of 5/15, and the genetically corrected iPSCs were differentiated into cardiomyocytes (iCMs). Importantly, the full-length dystrophin expression and membrane localization were restored in genetically corrected iPSCs and iCMs. Thus, this is the first study demonstrating that full-length dystrophin can be restored in iPSCs and iCMs via targeted exon addition, indicating potential clinical prospects for DMD gene therapy.

## 1. Introduction

Duchenne muscular dystrophy (DMD) is an X-linked recessive progressive muscle disease caused by mutations in the *DMD* gene encoding dystrophin. It is the most common fatal genetic disease in childhood, with an estimated incidence of 1/3500–1/5000 male births. Patients with DMD are mainly characterized by dystrophin deficiency which leads to body-wide muscle degeneration, and even life-threatening cardiomyopathy, and up to 40% of patients may die from heart failure and/or sudden cardiac death [1]. Traditionally, DMD patients are treated with corticosteroids to delay the muscle atrophy progression, and there is currently no cure for DMD. A highly promising therapeutic strategy is gene therapy [2].

The ~2.4 Mb *DMD* encodes a ~14 kb cDNA with 79 exons and is the largest gene in the human genome. The size of the *DMD* coding sequence far exceeds the normal packaging capacity of viral vectors, which hampers exogenous gene delivery. Some researchers have reported the delivery of truncated dystrophin coding sequences via viral vectors or non-viral vectors [3,4]. Strategies such as splice site disruption via gene editing, exon skipping and stop codon read-through could enable DMD patients to achieve milder clinical symptoms and longer survival [5,6,7,8,9]. Eteplirsen, an antisense oligonucleotide (AON), is the first DMD therapeutic drug approved for clinical application by the US Food and Drug Administration (FDA). Eteplirsen can skip the frameshift exon 51 and restore the splicing, transcription and translation of the following exons, which may alleviate a severe DMD phenotype, with an effect similar to Becker Muscular Dystrophy (BMD). However, it cannot permanently restore the dystrophin level because it has a short half-life in vivo [10,11], and full-length dystrophin protein restoration remains challenging.

Homology-directed recombination (HDR)-based gene correcting is a well-established technique that has been widely used in gene therapy. A nonsense mutation within exon 23 of a DMD mice model (mdx) was corrected via HDR using ssODN in a previous study [12]. Although CRISPR/Cas9-mediated gene editing can permanently restore the reading frame, gene editing in vivo may bring about undesired genome editing outcomes, and the delivery of gene editing systems via Adeno-associated virus (AAV) also faces some obstacles, such as delivery efficiency and specificity, pre-existing immunity against AAV capsids, as well as vector-induced immune responses [13].

Ex vivo gene therapy strategy may possess higher safety than in vivo due to the reliability of cell sourcing by selecting cells without off-target mutations via whole-genome sequencing analysis [14]. Induced pluripotent stem cells (iPSCs) have become an ideal cell source for ex vivo gene therapy and autologous transplantation due to their unlimited proliferation ability and multi-directional differentiation potential. The genetic correction of DMD patient-derived iPSCs (DMD-iPSCs) and differentiation into suitable cells for transplantation holds promise for DMD gene therapy [15]. Studies have mostly validated the exon splicing strategy or delivery of mini-dystrophin in iPSCs [16,17] to restore the expression of partially deleted dystrophin, which could normalize skeletal muscle force but only partially correct electrocardiogram and heart hemodynamics [18]. HDR-based gene correction is an effective approach to restore the full-length dystrophin expression [19].

In this study, an in situ genetic correction approach to restore the full-length dystrophin protein coding region in DMD-iPSCs was developed via targeted addition of the missing exon using CRISPR/Cas9. Thus, dystrophin expression and localization were restored in the genetically corrected iPSCs and their derived cardiomyocytes (iCMs), indicating the potential clinical prospects of this in situ genetic correction strategy for DMD gene therapy.

## 2. Results

### 2.1. Design and Construction of CRISPR/Cas9 and Donor Template for In Situ Correction of DMD Mutations

**Figure 1:**
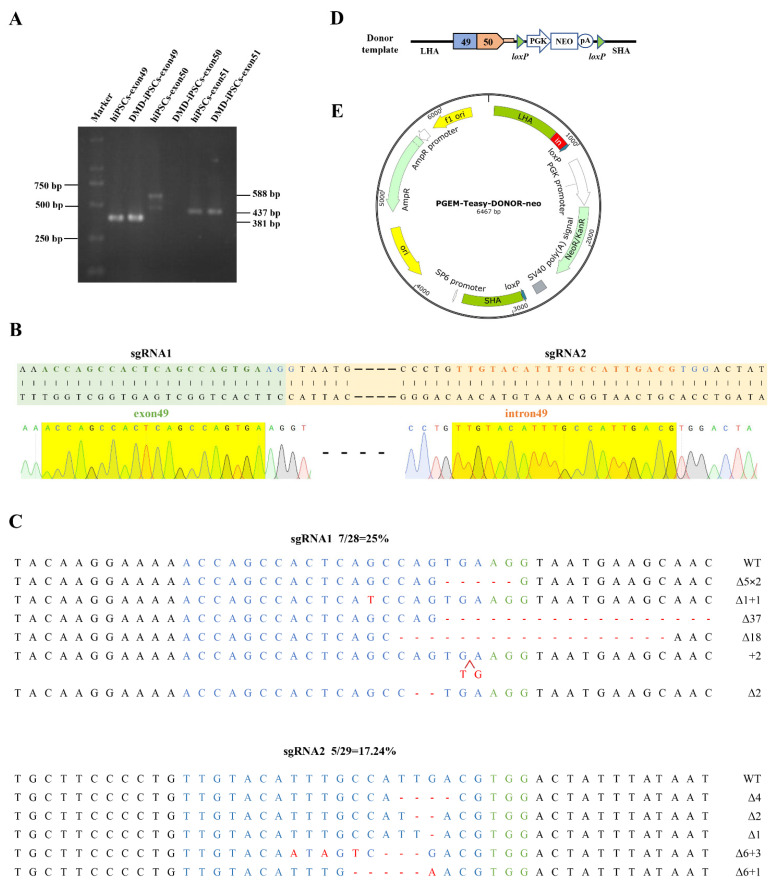
Gene editing components for DMD correction in situ. (**A**) Exons 49, 50 and 51 of DMD in DMD-iPSCs were amplified by PCR, and the deletion of exon 50 in DMD-iPSCs was verified using agarose gel electrophoresis. (**B**) sgRNAs targeting DMD. The green-shaped is exon 49, the green font indicates the sgRNA sequence targeting the 3′ end of exon 49, the yellow-shaped part is intron 49, and the yellow font indicates the sgRNA sequence targeting the 5′ end of intron 49, the blue font indicates the PAM sequences. The yellow shadows below are the Sanger sequencing results of the two sgRNAs, and in our all Sanger sequencing results, the green, red, blue and black font and sequencing map represent the base A, T, C, G respectively. (**C**) Detection of CRISPR/Cas9 efficiency by TA cloning. WT, wild-type; ∆, deletion; +, insertion; ×, times. The red arrow indicates the position of insertion. The green-labeled bases are the PAM sequences of CRISPR/Cas9, the target sequence of sgRNA is shown in blue, and red indicates inserted bases. (**D**,**E**) Schematic diagrams of the donor template and plasmid. The donor template includes the ~700 bp long homology arm (LHA) containing DMD exon 49, DMD exon 50 with its ~50 bp splice donor (SD) at the 5’ end of intron 50, a PGK-Neo selection box that can be removed by cre digestion and the ~700 bp short homology arm (SHA) following the cleavage site. The donor template was loaded into the pGEM T easy vector plasmid.

We previously generated an iPSC line derived from the urine cells of a DMD patient with exon-50-deleted (DMD-iPSCs) [20] that was detected via multiplex ligation-dependent probe amplification (MLPA). Here, exons 49, 50 and 51 in DMD-iPSCs were identified using PCR-amplification, with the exon-spanning primers (Table S1) and normal human iPSCs (hiPSCs) as a positive control (Figure 1A). In order to insert the deleted exon 50 in situ, we designed two single-guide RNAs (sgRNAs) to target the 3’ end of exon 49 (sgRNA1) and the 5’ end of intron 49 (sgRNA2) (Figure 1B, Table 1), and verified the cleavage activity with a frequency of 25% and 17.24%, respectively, via Sanger sequencing (Figure 1C). The sgRNA1 was used for the subsequent experiment, and the donor template plasmid construction was based on the corresponding site.

We used the genomic DNA (gDNA) of hiPSCs as a template to amplify the exon 50 of DMD and its splicing donor sequence, and the gDNA of DMD-iPSCs as a template to amplify the homology arms (Table S2). These fragments were ligated and constructed into the T easy vector. In order to screen the site-specific integrated cells efficiently, the neomycin resistance cassette (Neo) flanked by LoxP sites was further inserted into the donor plasmid (Figure 1D,E). The donor plasmid was identified via Sanger sequencing (Figure S1).

### 2.2. CRISPR/Cas9-Mediated DMD In Situ Correction in DMD-iPSCs

**Figure 2:**
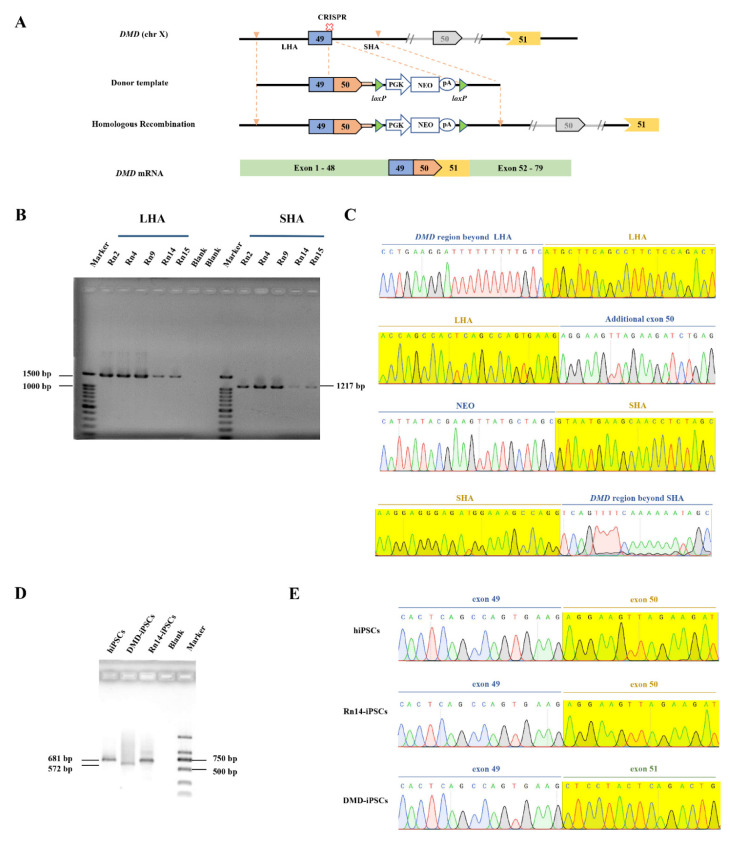
In situ correction of *DMD* in DMD-iPSCs. (**A**) Schematic illustration of in situ correction. Exon 50 and the neo expression cassette were inserted at the targeted cleavage site by homologous recombination to restore intact *DMD* transcripts by RNA splicing. (**B**,**C**) Positive clones were identified by PCR and Sanger sequencing. The yellow shadows indicate the homology arm. (**D**,**E**) Transcription level identification by PCR and Sanger sequencing. Reverse transcription PCR (RT-PCR) analysis of Rn14-iPSCs with primers spanning DMD exons 49–54, with hiPSCs and DMD-iPSCs as controls. The yellow shadows indicate the exon 50 or exon 51. In all Sanger sequencing results, the green, red, blue and black font and sequencing map represent the base A, T, C, G respectively.

The CRISPR/Cas9 and the donor template plasmid were co-nucleofected into DMD-iPSCs (Figure 2A), followed by G418 drug screening after 48 h for 3 days. The cells that survived after G418 treatment were digested into single cells for monoclonal formation. Fifteen single clones were picked up and subjected to PCR amplification with primers across the homology arms (~1.5 kb across LHA and ~1.1 kb across SHA). The expected band was detected among 5 out of 15 clones (33.33%) by agarose gel electrophoresis Figure 2B and Figure S2). Sanger sequencing was performed to verify DMD-repaired iPSCs (Figure 2C). One stably genetically corrected clone (Rn14-iPSCs) was selected for further research. The total RNA of Rn14-iPSCs was extracted, and the transcripts were identified by primers across exons 49–54. The RT-PCR showed that Rn14-iPSCs were identical to hiPSCs with a 681 bp band, while DMD-iPSCs lacking exon 50 were identical to a 572 bp band (Figure 2D). The sequencing results revealed that exon 50 was successfully inserted into DMD in Rn14-iPSCs with its splicing and transcription restored (Figure 2E).

**Figure 3:**
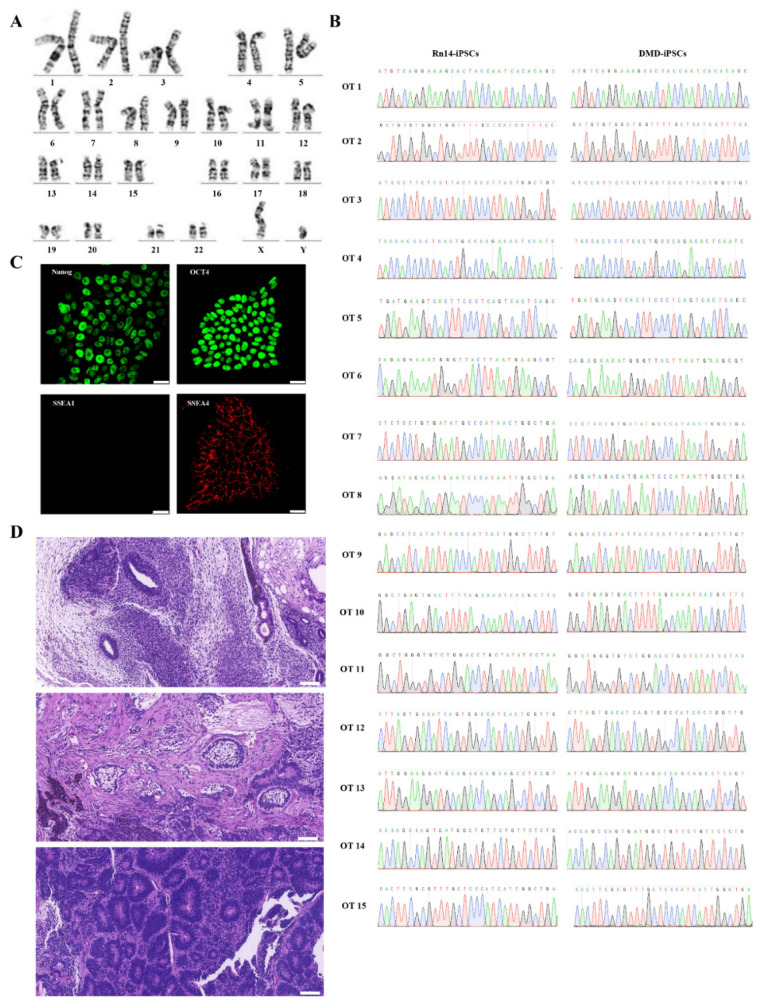
Identification of Rn14-iPSCs. (**A**) Karyotype analysis results showed normal karyotype of Rn14-iPSCs. (**B**) Sanger sequencing of 15 potential off-target sites after targeted gene correction in Rn14-iPSCs; no indels were detected compared to DMD-iPSCs at these sites. The green, red, blue and black font sequencing map represent the base A, T, C, G respectively. (**C**) Immunostaining of Rn14-iPSCs with pluripotency markers NANOG (green), OCT4 (green), SSEA-1 (red) and SSEA-4 (red). Nuclei were stained using DAPI. Scale bar: 25 µm. (**D**) H&E staining of Rn14-iPSCs-derived teratoma confirmed the potential of Rn14-iPSCs to form the three germ layers (ectoderm, mesoderm and endoderm). Scale bar: 100 µm.

Further analysis showed that the Rn14-iPSCs were karyotypically normal (Figure 3A). The gDNA of DMD-iPSCs and Rn14-iPSCs were extracted, PCR-amplified and Sanger sequenced to evaluate potential off-target activity of CRISPR/Cas9. The top 15 potential off-target sites predicted using Cas-OFFinder, which is accessible at http://www.rgenome.net, (accessed on 1 June 2021) were detected, and the results revealed that no indels were found, as compared with DMD-iPSCs (Figure 3B, Table S3). Additionally, Rn14-iPSCs maintained pluripotency markers (OCT4, NANOG and SSEA4) expression and no differentiation marker SSEA-1 expression (Figure 3C). The teratomas derived from Rn14-iPSCs in vivo showed that the Rn14-iPSCs could differentiate into all three germ layers (Figure 3D), indicating that the targeted addition of exon 50 did not affect pluripotency.

### 2.3. Dystrophin Expression in the Rn14-iPSCs-Derived Cardiomyocytes

**Figure 4:**
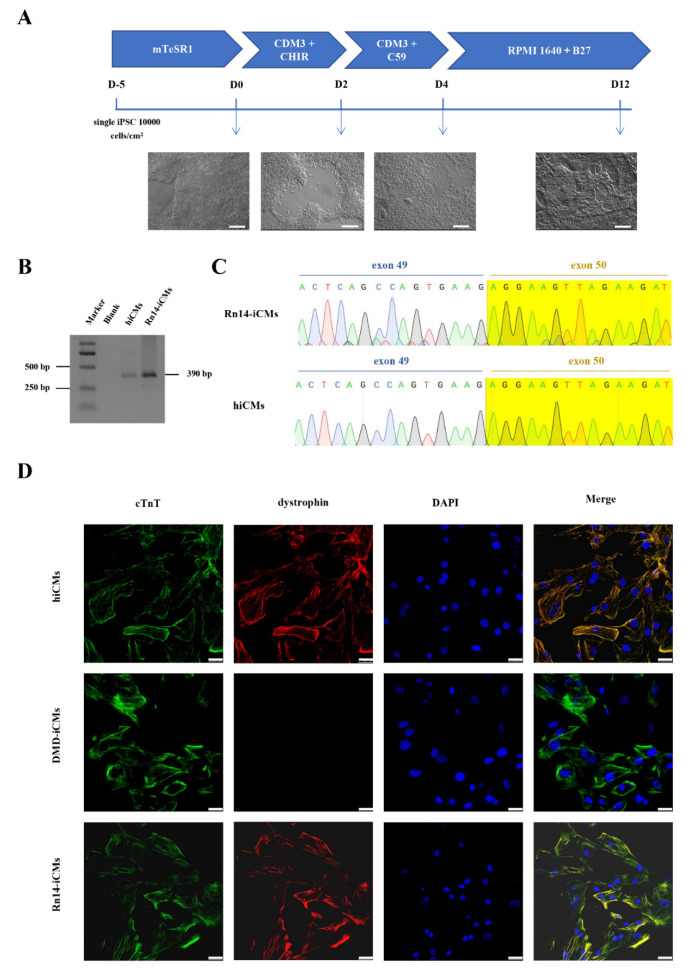
Expression of in situ corrected *DMD* in Rn14-iPSCs-derived cardiomyocytes (Rn14-iCMs). (**A**) Schematic diagram of the protocol for directional induction of iPSCs into cardiomyocytes and dynamic changes of cell morphology during differentiation of iPSCs into cardiomyocytes (iCMs). Scale bar: 200 μm. (**B**,**C**) RT-PCR analysis and Sanger sequencing with primers spanning *DMD* exon 50 of Rn14-iCMs and hiCMs were identical, with exon 50 existing at the cardiomyocyte stage. The green, red, blue and black font sequencing map represent the base A, T, C, G respectively. (**D**) Immunostaining for cardiomyocyte-specific markers cardiac troponin T (cTnT) (green) and dystrophin (red), and where the two markers merged were shown in yellow. DAPI was used to visualize the nucleus (blue). Scale bar: 25 µm.

In DMD, damaged cardiomyocytes which lead to cardiomyopathy finally cause the mortality of DMD patients. To verify that the full-length dystrophin expression was restored in cardiomyocytes, we differentiated the genetically corrected Rn14-iPSCs, DMD-iPSCs, and normal control hiPSCs into cardiomyocytes (Rn14-iCMs, DMD-iCMs and hiCMs) using a small molecule-induced protocol (Figure 4A). During the differentiation, the morphology of all the three iPS cell lines gradually changed from stem cell clones with smooth edges to triangular or spindle-shaped muscle-like cells, following with interweaving and beating spontaneously 10 days later.

RT-PCR and sequencing with primers spanning DMD exon 50 indicated that Rn14-iCMs and hiCMs were detected with the exon 50-corrected dystrophin transcripts (Figure 4B,C). All iCMs expressed the cardiomyocyte-specific marker cardiac troponin T (cTnT) (Figure 4D) and exhibited spontaneous contractions (Videos S1–S3). More importantly, we verified the full-length dystrophin restoration via immunofluorescence using a C-terminal mouse anti-dystrophin (7A10) antibody corresponding to amino acids 3558–3684 of dystrophin. Immunofluorescence showed abundant dystrophin staining signals in Rn14-iCMs and hiCMs, and very weak and rare signals in DMD-iCMs (Figure 4D). These results suggest that full-length dystrophin expression and localization was restored at the cardiomyocyte stage.

## 3. Discussion

Most of the previous studies have focused on exon skipping, and the results demonstrated that severe DMD phenotypes could be alleviated. Some gene-editing strategies were also employed to produce *DMD* transcripts with partial exons deleted by disrupting exon splicing sites. Unless large fragments that cover several exons for different mutations are skipped, a specific editing tool is still necessary [9]. AAV-mediated mini-dystrophin gene delivery, which can cover all *DMD* mutations, has been reported recently, and some have shown promising results in preclinical studies; however, there are still some immune and safety issues that need to be addressed in clinical trials. In a long-term follow-up study of AAV-based HA gene therapy, AAV vectors were found to be integrated near genes that control cell growth in hepatocytes of a HA canine model, indicating a potential risk of tumorigenicity [21]. In another clinical trial (NCT03199469) of AAV-mediated X-linked myotube myopathy, two children died of sepsis, and potential high-dose AAV toxicity was also observed in some subjects enrolled in the DMD gene therapy trials [22]. In addition, the mainstream version of the human mini-dystrophin gene currently used has achieved restoration of most skeletal muscle function, as well as production of abnormal electrocardiogram and heart hemodynamics due to the absence of spectrin repeats 16 to 19 (R16-19). Moreover, all electrocardiogram abnormalities and the end-diastolic volume in a 23-month-old mouse model of Duchenne Dilated Cardiomyopathy were normalized via the cardiac-specific expression of ∆H2-R15 mini-dystrophin, but this caused cardiac hypertrophy, and the size of this mini-dystrophin gene is nearly 7 kb, which is too large for packaging into the AAV capsids [23]. Numerous studies have shown that the absence of full-length dystrophin resulted in slower relaxation kinetics, reduced myofibril contractile tension, and abnormal Ca^2+^ handling, suggesting delayed maturation and altered structures of cardiomyocytes associated with these functions [24,25]. A distinct pool of dystrophin was co-localized with α-actin and desmin in the Z-disc of cardiac myofibrils, but not in skeletal muscle, and the significant pool of the 427 kDa form of cardiac dystrophin was specifically associated with the contractile apparatus at the Z-discs [26]. The association of dystrophin with cardiomyocyte T tubules is exclusively seen in excitation−contraction coupling and not in the transmission of contractile force [27], suggesting that dystrophin may play diverse roles in cardiomyocytes. These results indicate that the function of *DMD* needs to be further explored due to the complexity of the gene, and the treatment of DMD cannot bypass the cardiomyocytes rescue and full-length dystrophin restoration.

Here, we precisely corrected the mutation in situ through the CRISPR/Cas9 and donor plasmid in patient-derived iPSCs, and no detectable off-target indels were found at the predicted sites using Sanger sequencing. The off-target activity of our editing system remains to be further determined using whole genome sequencing before therapeutic application [28,29]. Compared to ssODN-mediated HDR, the targeted addition can cover larger fragments of mutation. Simultaneously, gene editing in situ can maximize the retention and persistence of transcripts of the corrected *DMD*, while the AONs used in the exon skipping strategy have a short half-life and lead to the failure of the treatment. Moreover, genetically corrected iPSCs in vitro have multi-directional differentiation potential, which can be differentiated into various cell types, including cardiomyocytes.

In fact, DMD is also a disease caused by stem cell defect. Skeletal muscle has significant regenerative potential, and muscle-resident stem cells, myogenic progenitor cells (MPCs) and especially muscle satellite cells (SCs) play a key role in muscle regeneration and DMD progression prevention, helping them to replenish the progenitor cell pool. Previous studies have confirmed that dystrophin expression was detected in the muscle of mdx mice after the transplantation of MPCs in the tibialis anterior muscle, and transplantation of freshly isolated SCs could reconstitute muscle fibers and quiescent SCs [19,30,31,32]. However, it is hardly to obtain a sufficient quantity of functional SCs for transplantation due to the great difficulty of isolating SCs, and the reduced ability to regenerate and transplant after culturing in vitro. Employing genetically corrected patient-specific iPSCs as a source of SCs is expected to cure DMD via autologous transplantation [33]. To achieve higher muscle engraftment rates in vivo, allowing transplanted cells to cross the muscle endothelial barrier and fuse with regenerating muscle fibers while avoiding the abnormal aggregation of high concentrations of transplanted cells in vascularized organs such as the liver and lung, many studies have reported synthetic bioadhesive hydrogels co-delivered with Wnt7a and MuSCs to enhance cell regeneration and migration in mouse muscle [34], or enhanced SCs migration by small-molecule DLL4 and PDGF-BB treatment [35]. P38 inhibition can keep isolated activated human SCs in an undifferentiated state, and maintain proliferation ability, while preserving the myogenic differentiation potential and improving the efficiency of intramuscular transplantation [36]. These advances make it possible to rescue muscle stem cell populations. However, it is worth noting that most of these strategies work locally in transplantation, while at least 30% to 50% of cardiomyocytes need to be repaired to rescue contractile dysfunction [9].

The human cardiomyocytes derived from iPSCs (hiPSC-CMs) provide an unlimited source of cells with uniform quantity and quality for transplantation. Here, we differentiated the iPSCs into cardiomyocytes and verified the restoration of full-length dystrophin expression and membrane localization. Furthermore, all iCMs exhibited spontaneous contractions. We observed that Rn14-iCMs and hiCMs showed higher spontaneous beating rates than DMD-iCMs (Videos S1–S3). The dystrophin-related myocardial function and the comparison at ultrastructural and electrophysiological levels will be investigated in further studies, while the further functional verification of cardiomyocytes is required before transplantation, such as the detection of the co-localization of dystrophin and DAPC complexes, and the establishment of 3D-engineered myocardium for the detection of myocardial physiology and function. It is inspiring that the allogeneic transplanted hiPSC-CMs have been confirmed to survive for 12 weeks and regenerate primate hearts, with no evidence of immune rejection. Moreover, the iPSC-CMs showed electrical coupling with host cardiomyocytes and improved cardiac contractile function at 4 and 12 weeks after transplantation [37].

Mesenchymal stem cells (MSCs) derived from iPSCs have great application value for the treatment of many diseases, including DMD. MSCs are low-immunogenic and have strong renewal ability and multi-directional differentiation potential. After skeletal muscle injury, the MSCs, transplanted via intravenous injection, can directly differentiate into skeletal muscle cells and secrete a variety of cytokines and regulatory factors to participate in the repair and regeneration of skeletal muscle, which significantly improves their function for 12 weeks after injection without severe side effects [38,39,40]. Methods for the prolonged engraftment of allogeneic MSCs have recently been reported, and the further development of these methods may allow the long-term cell engraftment of allogeneic MSCs [41]. Moreover, the co-transplantation of syngeneic MSCs improved the survival of allografted iPSC-CMs [42]. We believe that iSCs and iMSCs as well as iCMs will be promising cell types for DMD transplantation therapy.

All in all, our study demonstrated that full-length dystrophin was restored in iPSCs and iCMs via targeted exon addition, indicating potential clinical prospects for DMD gene therapy with advances in cell transplantation therapy.

## 4. Materials and Methods

### 4.1. Cell Culture

The normal hiPSCs (DYR0100) were purchased from ATCC. DMD-iPSCs were generated previously [20]. Briefly, DMD-iPSCs were reprogrammed from urine cells of a severe DMD patient with exon 50 deletion. All iPSCs were routinely cultured (37 °C 5% CO_2_) on Matrigel (BD Biosciences, Franklin Lakes, NJ, USA) coated 12-well plates (Corning, New York, NY, USA) in mTesR1 medium (StemCell Technologies, Vancouver, BC, Canada).

The HEK 293T cells were routinely cultured (37 °C, 5% CO_2_) on 6-well plates (Corning) in DMEM basic medium (Gibco, New York, NY, USA) containing 10% FBS (Gibco), 2 mM GlutaMAXTM (Gibco).

### 4.2. Construction of CRISPR/Cas9 Plasmids and Detection of Cleavage Activity

Two sgRNAs-targeted human DMD exon 49 or intron 49 were designed using Optimized CRISPR Design (http://zlab.bio/guide-design-resources/) (accessed on 9 November 2020) [43], then the sgRNAs were verified via the CHOPCHOP website (http://chopchop.cbu.uib.no/) (accessed on 9 November 2020) [44] and CRISPR RGEN tool (http://www.rgenome.net/cas-designer/) (accessed on 9 November 2020) [45]. The primers of the sgRNAs were synthesized by Sangon Biotech (Shanghai, China), then annealed and cloned into pX330, which was a gift from Feng Zhang’s laboratory (Addgene No. 42230, Watertown, MA, USA). Briefly, the annealed sgRNAs and the BbsI (New England Biolabs, Ipswich, MA, USA) digested pX330 plasmid were ligated using T4 DNA ligase (Thermo Fisher Scientific, Waltham, MA, USA). For cleavage activity detection,1.5 µg CRISPR/Cas9 plasmids with sgRNA were transfected to 1.5 × 10^6^ HEK 293T cells with lipo2000 (Thermo Fisher Scientific), and pMaxGFP plasmid was used as a control. After 72 h, gDNA of the transfected cells was extracted for PCR amplification and T-A clone detection with the primers of the sgRNA target site: DMD-exon 49-F: 5′-GTGCCCTTATGTACCAGGCA-3′/DMD-exon 49-R: 5′-AAGACAGCTTTGCCTCTGCT-3′. The PCR products were ligated to the pGEM-T Easy vector (Sigma-Aldrich, St. Louis, MO, USA) and then transformed into DH5α competent (Thermo Fisher Scientific) for blue-white screening. Single white colonies were picked and sequenced by Sanger sequencing.

### 4.3. Construction of a Donor Vector for Gene Correction

A 708 bp long homology arm with an MfeI site and a 652 bp short arm with a NheI site were amplified from DMD-iPSCs gDNA. The coding sequence of exon 50 plus a splice donor was amplified from the hiPSCs gDNA. Then the three fragments were ligated to a pGEM-T Easy vector with homologous recombination kit ClonExpress II one-step cloning kit (Vazyme, Nanjing, China). A NheI flanked PGK-Neo cassette was amplified from previously constructed plasmid [46] and then inserted into NheI (New England BioLabs) linearized donor vector. All the sequences of primers were provided in Table 1.

### 4.4. In Situ Gene Correction in DMD-iPSCs and Off-Target Analysis

The DMD-iPSCs were nucleofected with the Human Stem Cell Nucleofector^®^ Kit 2 (LONZA, Walkersville, MD, USA) set at program B016 according to the manufacturer’s instructions. Briefly, the DMD-iPSCs were dissociated into single cells using TrypLE™ Express (Thermo Fisher Scientific) and counted. For 1.5 × 10^6^ cells, 5 µg of CRISPR/Cas9 plasmid and donor plasmid were used, respectively, to transfect the DMD-iPSCs, then the transfected cells were cultured in mTesR1 medium with 10 µM of Y27632 (STEMCELL Technologies). Two days later, 50 µg/mL of G418 (Sigma-Aldrich) was used for screening the gene targeted cells. The cells survived from the G418 selection were counted, and 1000 cells were seeded on Matrigel-coated 6 cm dishes and cultured in mTesR1 medium with CloneR (STEMCELL Technologies) for 8 to 10 days. Then individual clones were picked, expanded and screened by PCR.

To analyze the potential off-target effect of CRISPR/Cas9, the predicted off-target sites with Cas-OFFinder [47], which is accessible at http://www.rgenome.net. (accessed on 1 June 2021), were detected via Sanger sequencing. We isolated gDNA of hiPSCs and Rn14-iPSCs for the PCR amplification and Sanger sequencing of the top15 potential off-target sites.

### 4.5. Karyotyping

After being treated with 0.1 μg/mL colcemid (Sigma-Aldrich) for 2.5 h, the iPSCs were blocked at metaphase. Then the cells were harvested, pelleted and then incubated in 0.075 M KCl at 37 °C for 7 min. After fixation with Carnoy’s fixative (methanol: acetic acid = 3: 1, Sinopharm Chemical Reagent Co., Ltd., Shanghai, China), the spread of metaphase chromosome was performed using air drying. After being dropped onto glass slides, the chromosomes were G-banded with a Giemsa solution (Sigma-Aldrich) after appropriate baking at 75 °C and digestion with trypsin.

### 4.6. Characterization of iPSCs

Cells seeded on 24-well chamber slides were fixed in 4% paraformaldehyde for 20 min and permeabilized with 0.1% Triton-X 100 (Sigma-Aldrich) in DPBS (Thermo Fisher Scientific) for 15 min at room temperature. After blocking with 5% bovine serum albumin (R&D Systems, Minneapolis, MN, USA) for 30 min, the cells were incubated with the primary antibody (OCT4, ab181557, Abcam, Cambridge, UK; NANOG, ab109250, Abcam; SSEA-1, SCR001, Merck Millipore, Watford, UK; SSEA-4, SCR001, Merck Millipore) for 1 h at room temperature. After thorough washing, samples were blocked for 30 min and treated with corresponding secondary antibodies (BD Biosciences) in the dark for 1 h. Nuclei corresponded with 4, 6-Diamidino-2-phenylindole (DAPI). The stained cells were analyzed with confocal photography (Leica DM IRB, Wetzlar, Germany).

For teratoma formation, 5 × 10^6^ iPS cells in 140 μL DMEM/F12 and 70 μL Matrigel were injected into the groins of immunocompromised mice. Two months later, the formed teratomas were harvested and fixed in 4% paraformaldehyde, then sectioned and stained with H&E. The care and use of the animals complied with the guidelines of the Ethics Committee of the School of Life Sciences of Central South University. This study was approved by the Institutional Animal Care and Use Committee of the Center for Medical Genetics of Central South University.

### 4.7. RT-PCR

Total RNA was extracted using Trizol reagent (Sigma-Aldrich), then was reverse-transcripted with HiScript II RT SuperMix for qPCR (Vazyme) according to the manufacturer’s instructions.

For RT-PCR, primers RT-PCR-49-F: 5′-AGCAGTTCAAGCTAAACAACCGG-3′/RT-PCR-54-R: 5′-CCTAAGACCTGCTCAGCTTCTTC-3′ were used for iPSCs, and primers RT-PCR-50-F: 5′-AAAAGACCTTGGGCAGCTTG-3′/RT-PCR-50-R: 5′-ACCACAGGTTGTGTCACCAG-3′ for iCMs.

### 4.8. iPSC-CM Differentiation

As the method previously described [48], iPSCs were dissociated to aggregates by 0.5 mM EDTA (Thermo Fisher Scientific) and seeded on a Matrigel-coated 6-well plates in mTesR1 containing 10 µM Y27632 (STEMCELL Technologies). The medium was refreshed daily with mTesR1. After 5 days, confluence reached ~90%, the medium was changed to CDM3 medium (including L-ascorbic acid 2-phosphate (Wako Chemicals USA, Richmond, VA, USA) and recombinant human albumin (Sciencell Research Laboratories, Carlsbad, CA, USA) dissolved in the RPMI basal medium (Gibco)) supplemented with 4 μM CHIR99021 (Selleck, Houston, TX, USA) for two days, then the media was switched to CDM3 medium supplemented with 2 μM WNT-C59 (Selleck) for two days. Subsequently, the cells were cultured in the RPMI 1640 basal medium supplemented with B27-supplement (Thermo Fisher Scientific) for 8 days, during which spontaneous contraction can be observed. The medium was changed every other day during the differentiation.

### 4.9. Immunofluorescence Staining for iCMs

Cells were fixed in cold acetone for 10 min and incubated with primary antibodies (mouse anti-dystrophin (7A10), Santa Cruz Biotechnology, Dallas, TX, USA; anti-cardiac, Abcam, Cambridge, UK) overnight at 4 °C and secondary antibodies (BD Biosciences) in the dark for 1 h at room temperature.

## Figures and Tables

**Table 1:** sgRNA sequences.

Target Site	Guide Sequence (5′-3′)	Sense Sequence (5′-3′)	Anti-sense Sequence (5′-3′)
exon 49	CCTTCACTGGCTGAGTGGCTGGT	CACCGACCAGCCACTCAGCCAGTGA	AAACTCACTGGCTGAGTGGCTGGTC
intron 49	TTGTACATTTGCCATTGACGTGG	CACCGTTGTACATTTGCCATTGACG	AAACCGTCAATGGCAAATGTACAAC

## Data Availability

All data supporting the reported result in this study can be found in the Supplementary Materials.

## Supplementary Materials

The following supporting information can be downloaded at: https://www.mdpi.com/article/10.3390/ijms23169176/s1.
