# Supplementary material for: Full-Length Dystrophin Restoration via Targeted Exon Addition in DMD-Patient Specific iPSCs and Cardiomyocytes
Source: Int J Mol Sci. 2022 Aug 16;23(16):9176. doi: 10.3390/ijms23169176 (PMC9409156; doi:10.3390/ijms23169176)
Supplement: Supplementary file 1 [file ijms-23-09176-s001.zip › supplementary.pdf]

Supplementary Material

Table S1. Primers used in the study.

Primer Name	Primer Sequence (5′-3′)	Used for the experiment of
DMD exon 49-F<br>DMD exon 49-R	GTGCCCTTATGTACCAGGCA<br>AAGACAGCTTTGCCTCTGCT	PCR for the exon 49 of DMD（381 bp）
DMD exon 50-F<br>DMD exon 50-R	AATTATGCCTGGAGAAAGGG<br>CTTGTGCTCTTCTCTTCCTT	PCR for the exon 50 of DMD（588 bp）
DMD exon 51-F<br>DMD exon 51-R	ATTGGCTCTTTAGCTTGTGT<br>TGTGTTTGCTGAGAGAGAAA	PCR for the exon 51 of DMD （437 bp）
LHA-F<br>LHA-R	CAAAGACATACAGTCATTCCG<br>GGCTCCAATAGTGGTCAGT	PCR for the site-specific clones screening of LHA ~1.5 kb
neo-SHA-F<br>neo-SHA-R	GGGCTTCGGAATCGTTT<br>AGAATCAGGTAACCTACTGCTAAC	PCR for the site-specific clones screening of SHA ~1.1 kb
RT-PCR-49-F<br>RT-PCR-54-R	AGCAGTTCAAGCTAAACAACCGG<br>CCTAAGACCTGCTCAGCTTCTTC	RT-PCR and Sanger sequencing in iPSCs stage（681 bp / 572 bp）
RT-PCR-50-F<br>RT-PCR-50-R	AAAAGACCTTGGGCAGCTTG<br>ACCACAGGTTGTGTCACCAG	RT-PCR and Sanger sequencing in iCMs stage（390 bp）

Table S2. Sequences of donor template fragment and primers for construction.

Fragment Name	Primer Name	Primer Sequence (5′-3′)	Fragment Sequence (5′-3′)
LHA 708 bp	DONOR-L-F<br>DONOR-L-R	ATGCTTCAGCCTTCTCCAG<br>caattgCTTCACTGGCTGAGTGGCT	ATGCTTCAGCCTTCTCCAGACTGAAGAAAAGCAAATAATAAACAAAATTAATCGTTCTTCAGAAGGCCTGTTTGACTCATTAATAAATAATAAGCTTTGATACTGTGCTTTAAGTGTTTACCCTTTGGAAAGAAAATAATTTTGACAGTGATGTAGAAATAATTATTTGATATTTATTTCAAAACAAAATTTATATCCAATACTAAACACAGAATTTTGTAAAACAATAAGTGTATAAAGTAAAATGAACATTAGGATTATTGAGATTATTGTAGCTAAAACTAGTGTTTATTCATATAAATTATGTTAATAAATTGTATTGTCATTATTGCATTTTACTTTTTTGAAAAGTAGTTAATGCCTGTGTTTCTATATGAGTATTATATAATTCAAGAAGATATTGGATGAATTTTTTTTAAGTTTAATGTGTTTCACATCTCTGTTTCTTTTCTCTGCACCAAAAGCTACATTTTTGTGCCCTTATGTACCAGGCAGAAATTGATCTGCAATACATGTGGAGTCTCCAAGGGTATATTTAAATTTAGTAATTTTATTGCTAACTGTGAAGTTAATCTGCACTATATGGGTTCTTTTCCCCAGGAAACTGAAATAGCAGTTCAAGCTAAACAACCGGATGTGGAAGAGATTTTGTCTAAAGGGCAGCATTTGTACAAGGAAAAACCAGCCACTCAGCCAGTGAAGcaattg (Add MfeI restriction site)
E50-SD 166 bp	DONOR-E50-F<br>DONOR-E50-R	cagccactcagccagtgaagAGGAAGTTAGAAGATCTGAGCT<br>ATTACgctagcgctagcACTTTTGAACAAATAGCTAGAGCCAAAG	AGGAAGTTAGAAGATCTGAGCTCTGAGTGGAAGGCGGTAAACCGTTTACTTCAAGAGCTGAGGGCAAAGCAGCCTGACCTAGCTCCTGGACTGACCACTATTGGAGCCTGTAAGTATACTGGATCCCATTCTCTTTGGCTCTAGCTATTTGTTCAAAAGTgctagc (Add nheI restriction site)
SHA 652 bp	DONOR-S-F<br>DONOR-S-R	AGTgctagcgctagcGTAATGAAGCAACCTCT<br>gccgcgaattcactagtgatCCTGGCTTTCCATCTCC	(Add nheI restriction site) gctagcGTAATGAAGCAACCTCTAGCAATATCCATTACCTCATAATGGGTTATGCTTCCCCTGTTGTACATTTGCCATTGACGTGGACTATTTATAATCAGTGAAATAACTTGTAAGGAAATACTGGCCATACTGTAATAGCAGAGGCAAAGCTGTCTTTTTGATCAGCATATCCTATTTATATATTGTGATCTTAAGGCTATTAACGAGTCATTGCTTTAAAGGACTCATTTCTGTCCTGGTGTGCTGCCATCAATACAAAAGTAGTCCCACCTTCAAGGTAGATTAAATTCTTTGGGGCTTTATTGCTTTGCTTGCCAGCCTTGATGCTTTTCATATTGTTTGGTTTAATTCAAATCAAGCTACTGCATCATAGTGTCTGTCTCCAACAGCTGTAAAGAATCACAATATGGTCCGTGACCTTTCTTTTCTTTGTTGCTCTTTCTTATAAAAGAGTAAAATCGGCATTGAGGCTTCAGTATTTTATTGGGTCAAGAATTTTTAGTGAAATGTCATGTAATTGCTTATTTCTTTGGCAAAAAGCTGGCCTGGGATAAACCTTTCTACTTTTTTTTCTTCTCCATATCCATGTTGAAGTCTAGGTCACATTTATTCACAGGCTATTACATAAGGAGGGAGATGGAAAGCCAGG

NEO<br>1949 bp	neo-F	CTAGCTATTTGTTCAAAGTgctagcATAACTTCGTATAGCATA	ATAACTTCGTATAGCATACATTATACGAAGTTATAC<br>ATGTCACAAAAGGAAACTCACCCTAACTGTAAAGT<br>AATTGTGTGTTTTGAGACTATAAATATCCCTTGGAG<br>AAAAGCCTTGTTAACGCGCGGTGACCCTCGAGTACT<br>AGGATCCATTAGGGAATTCGTCGACCTCGAAATTCT<br>ACCGGGTAGGGGAGGCGCTTTTCCCAAGGCAGTCTG<br>GAGCATGCGCTTTAGCAGCCCCGCTGGGCACTTGGC<br>GCTACACAAGTGGCCTCTGGCCTCGCACACATTCCA<br>CATCCACCGGTAGGCGCCAACCGGCTCCGTTCTTTG<br>GTGGCCCCTTCGCGCCACCTTCTACTCCTCCCCTAGT<br>CAGGAAGTTCCCCCCCGCCCCGCAGCTCGCGTCGTG<br>CAGGACGTGACAAATGGAAGTAGCACGTCTCACTA<br>GTCTCGTGCAGATGGACAGCACCGCTGAGCAATGG<br>AAGCGGGTAGGCCTTTGGGGCAGCGGCCAATAGCA<br>GCTTTGCTCCTTCGCTTTCTGGGCTCAGAGGCTGGGA<br>AGGGGTGGGTCCGGGGGCGGGCTCAGGGGCGGGCT<br>CAGGGGCGGGGCGGGCGCCCGAAGGTCCTCCGGAG<br>GCCCGGCATTCTGCACGCTTCAAAAGCGCACGTCTG<br>CCGCGCTGTTCTCCTCTTCCTCATCTCCGGGCCTTTC<br>GACCTGCATCCtctagaGCCACCATGATTGAACAAGAT<br>GGATTGCACGCAGGTTCTCCGGCCGCTTGGGTGGAG<br>AGGCTATTCGGCTATGACTGGGCACAACAGACAAT<br>CGGCTGCTCTGATGCCGCCGTGTTCCGGCTGTCAGC<br>GCAGGGGCGCCCGGTTCTTTTTGTCAAGACCGACCT<br>GTCCGGTGCCCTGAATGAACTGCAGGACGAGGCAG<br>CGCGGCTATCGTGGCTGGCCACGACGGGCGTTCCTT<br>GCGCAGCTGTGCTCGACGTTGTCACTGAAGCGGGAA<br>GGGACTGGCTGCTATTGGGCGAAGTGCCGGGGCAG<br>GATCTCCTGTCATCTCACCTTGCTCCTGCCGAGAAA<br>GTATCCATCATGGCTGATGCAATGCGGCGGCTGCAT<br>ACGCTTGATCCGGCTACCTGCCCATTCGACCACCAA<br>GCGAAACATCGCATCGAGCGAGCACGTACTCGGAT<br>GGAAGCCGGTCTTGTCGATCAGGATGATCTGGACGA<br>AGAGCATCAGGGGCTCGCGCCAGCCGAACTGTTCG<br>CCAGGCTCAAGGCGCGCATGCCCGACGGCGAGGAT<br>CTCGTCGTGACCCATGGCGATGCCTGCTTGCCGAAT<br>ATCATGGTGGAAAATGGCCGCTTTTCTGGATTCATC<br>GACTGTGGCCGGCTGGGTGTGGCGGACCGCTATCAG<br>GACATAGCGTTGGCTACCCGTGATATTGCTGAAGAG<br>CTTGGCGGCGAATGGGCTGACCGCTTCCTCGTGCTTT<br>ACGGTATCGCCGCTCCCGATTCGCAGCGCATCGCCT<br>TCTATCGCCTTCTTGACGAGTTCTTCTGAGCGGGACT<br>CTGGGGTTCGAAATGACCGACCAAGCGACGCCCAA<br>CCTGCCATCACGAGATTTCGATTCCACCGCCGCCTT<br>CTATGAAAGGTTGGGCTTCGGAATCGTTTTCCGGGA<br>CGCCGGCTGGATGATCCTCCAGCGCGGGGATCTCAT<br>GCTGGAGTTCTTCGCCCACCCCAACTTGTTTATTGCA<br>GCTTATAATGGTTACAAATAAAGCAATAGCATCACA<br>AATTTCACAAATAAAGCATTTTTTTCACTGCATTCTA<br>GTTGTGGTTTGTCCAAACTCATCAATGTATCTTATCA<br>TGTCTGTATACCGTCGACCTCTAGCTAGAGCTTGGC
	neo-R	GCTAGAGGTTGCTTCATTACgctagcTAACTTCGTATAATGTATG	

	GTAATCATGGTCATAGCTGTTTCCTGTGTGAAATTGT TATCCGCTCACAATTCCACACAACATACGAGCCGG AAGCAATAACTTCGTATAGCATACATTATACGAAGT TAgctagc (Add nheI restriction site)

Table S3. 15 potential off-target sites predicted by CAS-OFFinder and primers for identification.

	Number of mismatched bases；match values	location on the chromosome		primer(5′-3′)
	0；1	ontarget_mm0_exon_DMD_chrX_31836716	F	GTGCCCTTATGTACCAGGCA
			R	AAGACAGCTTTGCCTCTGCT
1	4；0.64	mm4_intron_GUCY2F_chrX_109374463	F	GCAAGTAACAGCAACAGAGGAGTG
			R	CTGACTCACTGACACCTGGGG
2	2；0.61	mm2_intergenic_RNU6-1078P|RGAG4_chrX_72073232	F	GGGAAGATGCGATGTGAGATAG
			R	TAGGCGAAAGGTGAGAGTTGGT
3	4；0.56	mm4_intergenic_MIR548AP|RP11-553E24.3_chr15_85947521	F	GGGATTAGGAGGCATTAGAAGA
			R	CAAGGATAGAGAAAAGGGGAAC
4	4；0.53	mm4_intron_GRK4_chr4_3030352	F	GTATTTTGAGGTGGGTAAGGGTT
			R	GGGACTTCTGTTCTATTTGCTGC
5	4；0.51	mm4_intron_CPLX2_chr5_175803726	F	AAAGAGAGGAGTGTGCCGAGG
			R	TTTATTTCCCAGCAGGTGAGTC
6	3；0.50	mm3_intergenic_CNTNAP4|AC010528.1_chr16_76356648	F	GAGACTGGGAAAGAGGCA
			R	AGCAGGGCTACTTCTCTCAA
7	4；0.45	mm4_intron_CTB-113P19.3_chr5_151762937	F	GTGAAACCTATTCAGTTAAAGTCCT
			R	GCAGGTTTGTCTGGGTTGTC
8	3；0.43	mm3_intron_SYNDIG1_chr20_24589161	F	TTTTGGAAGAAATGACCCCT
			R	TGTGCCCATACTTGCTACCT
9	4；0.39	mm4_intron_AC004691.5_chr7_30800412	F	TTTTTGTGCCCCTCTTAGTCAT
			R	TCCTGCCCCCTCAATAGC
10	4；0.39	mm4_intron_TMEM163_chr2_134497742	F	ACTCTGAATCCCAAGTGCCT
			R	TCAAGGTTGCCCCAAATAGT
11	4；0.37	mm4_intron_MPHOSPH8_chr13_19663742	F	GGCTTGTTCCTGTGTTCTGG
			R	CTGGCTGTGCTCTTTTGCTT
12	4；0.37	mm4_intergenic_RNU5D-2P|C9orf171_chr9_132423670	F	TTACAGATGGGGAAGCCGA
			R	GGTCCTTGGAGAACAGAAAACA
13	3；0.36	mm3_intergenic_CCDC185|CAPN8_chr1_223482694	F	AGAGACAGAAAGTTGAATGGTGG
			R	CAGGCAGTCTAATAGCAGTCCA
14	4；0.36	mm4_intergenic_MED10|UBE2QL1_chr5_6404252	F	GCTGGGATGTGTTTCTGGTG
			R	ATCTGGAATGAACAGTCTTGGG
15	4；0.35	mm4_intergenic_RP11-267C16.1|RP11-25O3.1_chr18_51961725	F	GTCCTGCTTTGGCATTCAT
			R	AGTAAGATAGGGGTGGGGG

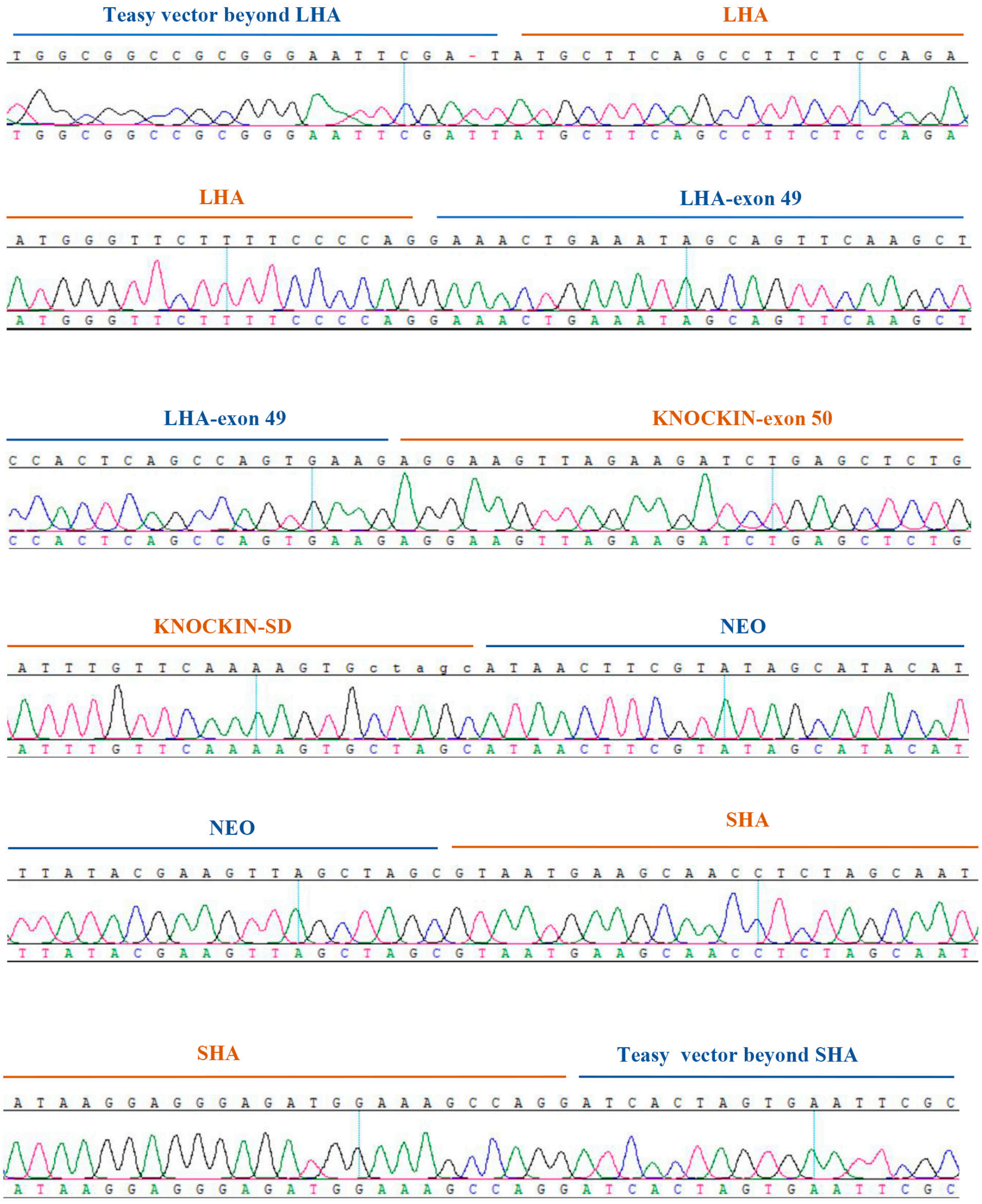

Figure S1. Sanger sequencing of the constructed donor vector.

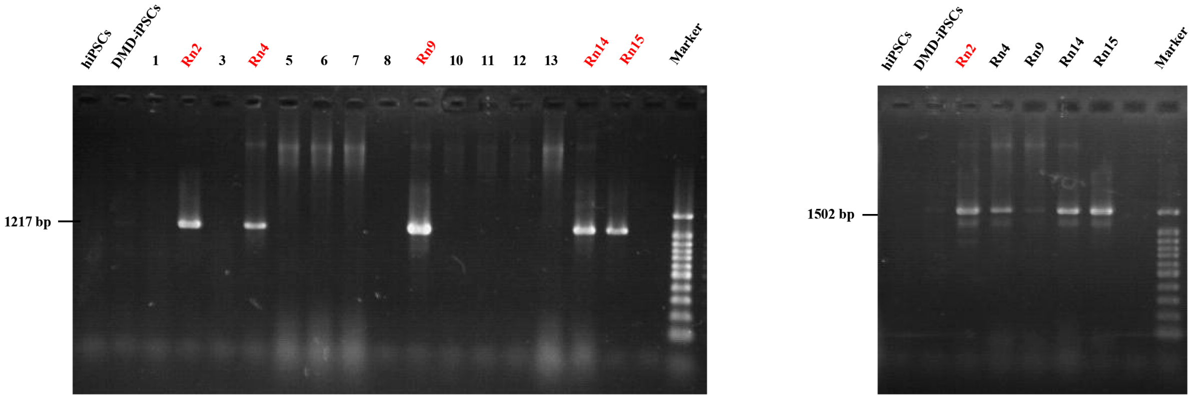

Figure S2. Identification of targeted clones by PCR pruducts using primers across the homology arms, hiPSCs and DMD-iPSCs were used as controls. The results of agarose gel electrophoresis showed that 5 (2, 4, 9, 14, and 15) out of 15 clones existed the predicted band.
